# Sinoatrial tissue of crucian carp heart has only negative contractile responses to autonomic agonists

**DOI:** 10.1186/1472-6793-10-10

**Published:** 2010-06-11

**Authors:** Matti Vornanen, Mervi Hälinen, Jaakko Haverinen

**Affiliations:** 1 University of Eastern Finland, Department of Biology, 80101 Joensuu, Finland

## Abstract

**Background:**

In the anoxia-tolerant crucian carp (*Carassius carassius*) cardiac activity varies according to the seasons. To clarify the role of autonomic nervous control in modulation of cardiac activity, responses of atrial contraction and heart rate (HR) to carbacholine (CCh) and isoprenaline (Iso) were determined in fish acclimatized to winter (4°C, cold-acclimated, CA) and summer (18°C, warm-acclimated, WA) temperatures.

**Results:**

Inhibitory action of CCh was much stronger on atrial contractility than HR. CCh reduced force of atrial contraction at an order of magnitude lower concentrations (EC_50 _2.75-3.5·10^-8 ^M) in comparison to its depressive effect on HR (EC_50 _1.23-2.02·10^-7 ^M) (P < 0.05) without differences between winter and summer acclimatized fish. Inhibition of nitric oxide synthase with 100 μM L-NMMA did not change the response of the sinoatrial tissue to CCh. Reduction of atrial force was associated with a strong shortening of action potential (AP) duration to ~50% (48 ± 10 and 50 ± 6% for CA and WA fish, respectively) and 11% (11 ± 3 and 11 ± 2% for CA and WA fish, respectively) of the control value at 3·10^-8 ^M and 10^-7 ^M CCh, respectively (P < 0.05). In atrial myocytes, CCh induced an inwardly rectifying K^+ ^current, I_K,CCh_, with an EC_50 _value of 3-4.5·10^-7 ^M and inhibited Ca^2+ ^current (I_Ca_) by 28 ± 8% and 51 ± 6% at 10^-7 ^M and 10^-6 ^M, respectively. These currents can explain the shortening of AP. Iso did not elicit any responses in crucian carp sinoatrial preparations nor did it have any effect on atrial I_Ca_, probably due to the saturation of the β-adrenergic cascade in the basal state.

**Conclusion:**

In the crucian carp, HR and force of atrial contraction show cardio-depressive responses to the cholinergic agonist, but do not have any responses to the β-adrenergic agonist. The scope of inhibitory regulation by CCh is increased by the high basal tone of the adenylate cyclase-cAMP cascade. Higher concentrations of CCh were required to induce I_K,CCh _and inhibit I_Ca _than was needed for CCh's negative inotropic effect on atrial muscle suggesting that neither I_K,CCh _nor I_Ca _alone can mediate CCh's actions but they might synergistically reduce AP duration and atrial force production. Autonomic responses were similar in CA winter fish and WA summer fish indicating that cardiac sensitivity to external modulation by the autonomic nervous system is not involved in seasonal acclimatization of the crucian carp heart to cold and anoxic winter conditions.

## Background

Crucian carp (*Carassius carassius *L.) is one of the most anoxia tolerant vertebrates and the only fish species in North temperate latitudes tolerates prolonged and total oxygen shortage. Therefore crucian carp thrive as dense single-species populations in seasonally anoxic ponds and lakes [1-3]. The success of crucian carp in inhabiting oxygen deprived environments is based on unsurpassed physiological adaptations to anoxia which involve huge carbohydrate stores throughout the body, anoxic metabolic depression, production of ethanol as an anaerobic end product, molecular mechanisms to allow anoxic brain survival, modification of gill structure to reduce ion loss in anoxia and inverse thermal compensation of heart function to minimize cardiac energy consumption in the anoxic and cold winter waters [4-8]. These adaptations are probably crucial for survival under severe hypoxia/anoxia up to 6 months in the ice covered lakes [6].

The vertebrate heart serves body homeostasis by distributing oxygen, metabolite substrates and hormonal messages throughout the body. In severely hypoxic or anoxic conditions, demands for oxygen convection diminish or completely disappear in the body of crucian carp, and due to a low metabolic rate in the cold, circulatory requirements for integrative functions are expected to reduce. Indeed, the heart of crucian carp shows clear seasonality in having larger glycogen reserves and fewer sarcolemmal (SL) L-type Ca^2+ ^channels and expressing lower myosin ATPase activity in winter than summer [3,9]. Furthermore, winter acclimatized fish show depressed SL Na^+ ^currents and increased K^+ ^currents in the heart [10-12]. These findings indicate that several intrinsic mechanisms of crucian carp cardiac myocytes are acclimatized to changing circulatory demands in seasonally varying habitat conditions and suggest that cardiac contractility is reduced in winter anoxia. However, the significance of extrinsic modulators of the heart, in particular the autonomic nervous regulation, is less well understood in the physiology of crucian carp and other anoxia-tolerant vertebrates.

In various physiological situations, contractility of the vertebrate heart is adjusted to circulatory demands by intrinsic length-dependent changes in individual myocytes [13,14] and by extrinsic modulators, mainly by the autonomic nervous system [15]. Vertebrate hearts usually have dual autonomic innervation through inhibitory cholinergic nerves and stimulatory adrenergic nerves, which modulate heart rate (HR), impulse conduction and force of contraction via specific membrane receptors and intracellular signaling cascades [16,17].

Noradrenaline and adrenaline of the sympathetic nervous system increase heart rate and force of contraction mainly by binding to beta-adrenergic receptors which use the cAMP-dependent pathway to increase SL Ca^2+ ^current (I_Ca_) and sarcoplasmic reticulum (SR) Ca^2+ ^uptake and release [17-19].

Acetylcholine (ACh) of the parasympathetic nervous system exerts a direct negative chronotropic effect on the sinoatrial nodal tissue, a negative dromotropic effect on atrio-ventricular conduction and a direct negative inotropic effect on the atria. Furthermore ACh has an indirect negative inotropic effect which is observed both in atrial and ventricular muscle in the presence of sympathetic tone. In isolated cardiac myocytes, muscarinic cholinergic agonists reduce and/or eliminate beta-adrenergic stimulation of the Ca^2+ ^current (I_Ca_), possibly by G-protein dependent inhibition of the adenylate cyclase. In the absence of beta-adrenergic stimulation, i.e. under basal conditions, ACh depresses I_Ca _if the adenylate cyclase is under a tonic activation [20,21].

Considering the drastic seasonal changes in abiotic conditions (temperature, oxygen content) of crucian carp habitat and associated alterations in functional demands on cardiac function, it could be anticipated that the extrinsic control mechanisms of the crucian crap heart might change according to the seasons. Therefore, the aim of the present study is to elucidate responses of the crucian carp sinoatrial preparations to sympathetic and parasympathetic agonists in fish acclimatized to winter and summer conditions. Specifically, it was hypothesized that negative parasympathetic responses are enhanced in winter and excitatory adrenergic effects are up-regulated in summer.

## Results

### Responses of the sinoatrial tissue to carbacholine (CCh)

**Figure 1:**
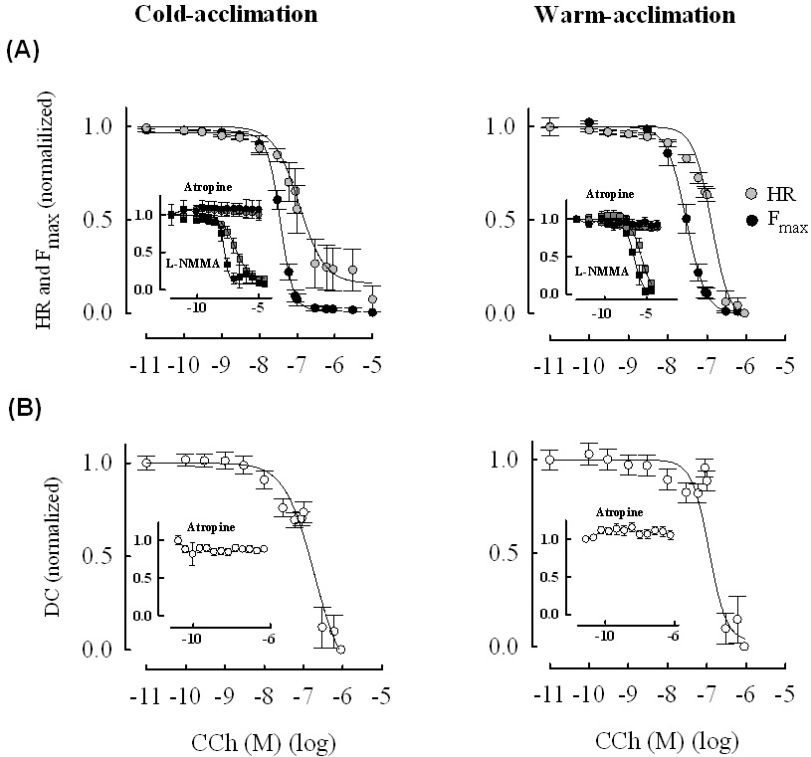
**Effects of CCh on contractility of crucian carp heart**. Concentration dependent effects of CCh on heart rate and atrial contractility in warm-acclimated and cold-acclimated crucian carp at the acclimation temperatures of the fish. (A) Depression of heart rate and force of atrial contraction (F_max_) by CCh. Note the difference in CCh sensitivity between heart rate and force of contraction. The inset indicates the effect of 3·10^-6 ^M atropine and 100 μM L-NMMA on the responses. (B) Effect of CCh on the duration of atrial contraction (DC). The inset shows blockade of the response in the presence of atropine. The results are means ± SEM of 15 and 19 fish for cold-acclimated and warm-acclimated fish, respectively.

Contractility of sinoatrial preparations was determined at the acclimation temperatures (4°C and 18°C) of the fish. Comparison of WA summer and CA winter fish hearts at basal state indicates a strong acute temperature effect on HR and kinetics of atrial contraction (Table 1). Cumulative additions of CCh to the bath caused a dose-dependent inhibition of HR and atrial force production in both acclimation groups (Figure 1). Force of atrial contraction was inhibited at low CCh concentrations and with similar EC_50 _values in WA (2.75·10^-8 ^M) and CA (3.5·10^-8 ^M) fish (P > 0.05). Inhibition of force and shortening of contraction duration appeared at the concentration of 10^-8 ^M CCh, but diverged slightly at higher CCh concentrations (P < 0.05) (Table 2). Inhibition of HR required almost an order of magnitude higher concentrations of CCh (2.02·10^-7 ^and 1.23·10^-7 ^M for CA and WA fish, respectively; P > 0.05) in comparison to its effect on atrial force production (P < 0.05). Both responses were completely abolished by 3·10^-6 ^M atropine, indicating that they were mediated by muscarinic cholinergic receptors (Figure 1, inset).

**Table 1:** Heart rate and variables of atrial contraction in sinoatrial preparations of thermally acclimated crucian carp

Variable	4°C acclimated fish	18°C acclimated fish
Heart rate (beats/min)	11.2 ± 0.5*	52.1 ± 1.7
Force (mN/mg)	1.2 ± 0.4*	3.4 ± 0.6
Time to peak force (ms)	774.3 ± 47.5*	237.4 ± 4.5
Time to half of relaxation (ms)	914.0 ± 45.2*	278.5 ± 8.2
Maximal rate of rise (mN/mg/ms)	0.04 ± 0.2*	0.21 ± 0.3
Maximal rate of fall (mN/mg/ms)	0.03 ± 0.1*	0.14 ± 0.2
Size of atrial tissues (mg)	6.71 ± 0.4	6.90 ± 0.6

Experiments were conducted at the acclimation temperature of the fish. The results are means ± SEM (n = 16-18). A statistically significant difference between acclimation groups is indicated by an asterisk (*, P < 0.05).

**Table 2:** EC_50_-values (concentration for half-maximal effect) of CCh for contractile and electrophysiological parameters of the crucian carp sinoatrial tissue and atrial cardiomyocytes

Parameter	4°C acclimated fish EC_50_	18°C acclimated fish EC_50_	Statistically significant differences (p < 0.05) between mean EC_50 _values with the listed parameters
			
HR	2.02e-7 ± 0.2	1.23e-7 ± 0.03	F_max_, IK
F_max_	3.50e-8 ± 0.03	2.75e-8 ± 0.1	HR, DC, IK
DC	1.11e-7 ± 0.1	1.46e-7 ± 0.1	F_max_, IK
IK, CCh at 4°C/18°C	4.47e-6 ± 0.2	3.47e-6 ± 0.7	HR, F_max_, DC

Taken together, these findings demonstrate a strong inhibitory effect of muscarinic cholinergic receptors on crucian carp heart and a large difference in sensitivity between chronotropic and inotropic responses to CCh.

### Effect of nitric oxidase synthase (NOS) inhibitor on CCh response

100 μM L-NMMA did not modify CCh's influence on HR and atrial contraction thus suggesting that NO generation by endocardial endothelial cells (or myocytes) is not involved CCh's negative chronotropic and inotropic effects in crucian carp sinoatrial tissue (Figure 1, inset).

### Responses of sinoatrial preparations to isoprenaline (Iso)

**Figure 2:**
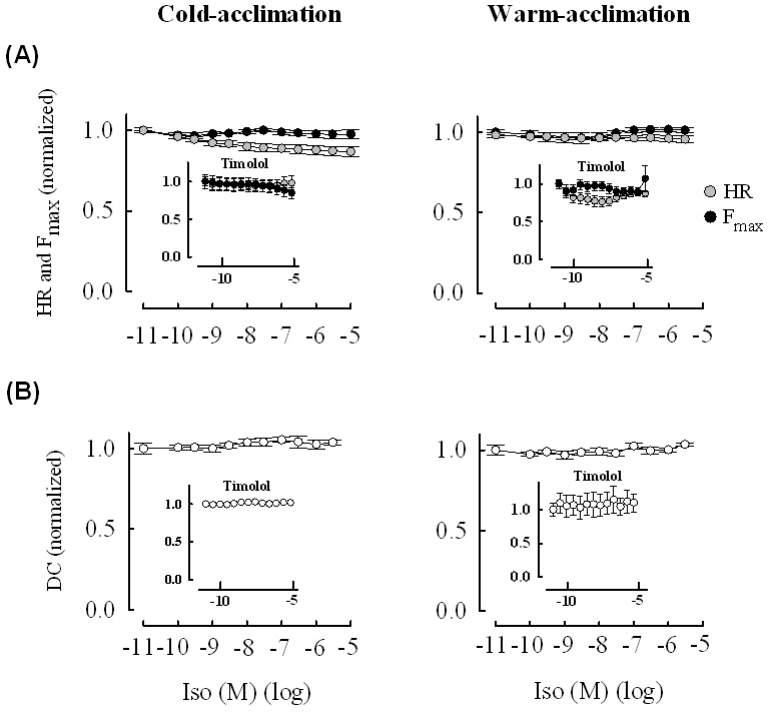
**Effects of Iso on crucian carp heart**. Concentration dependent effects of Iso on heart rate and atrial contractility in warm-acclimated and cold-acclimated crucian carp at the acclimation temperatures of the fish. Effect of Iso on heart rate and force of contraction (F_max_) (A) and on the duration of atrial contraction (DC) (B) in the absence and presence (inset) of 3·10^-6 ^M timolol. Note the absence of responses to Iso. Results are means ± SEM of 15 and 17 fish for cold-acclimated and warm-acclimated fish, respectively.

Sinoatrial preparations of the crucian carp heart were almost totally insensitive to the nonselective beta-adrenergic agonist Iso. Neither HR nor force of atrial contraction was changed by Iso (Figure 2). This suggests that i) either crucian carp heart lacks a functional beta-adrenergic cascade, or ii) signal transduction pathway mediating the beta-adrenergic effects is maximally activated under basal conditions or iii) Iso is activating antagonistic effects through beta and alpha adrenoceptors which cancel each other. However, the latter alternative can be excluded, since blockade of the beta-adrenergic receptors with 3·10^-6 ^M timolol did not reveal negative inotropic or chronotropic effects which would have cancelled the positive effects of beta-adrenergic stimulation (Figure 2, inset).

Collectively, these findings indicate negligible inotropic and chronotropic effects of beta-adrenergic stimulation in the crucian carp heart possibly due to the saturation of the signal transduction cascade under basal conditions (see *Effects of CCh on L-type Ca^2+ ^current of atrial myocytes*).

### Effects of CCh on action potential (AP) and K^+ ^currents of atrial myocytes

**Figure 3:**
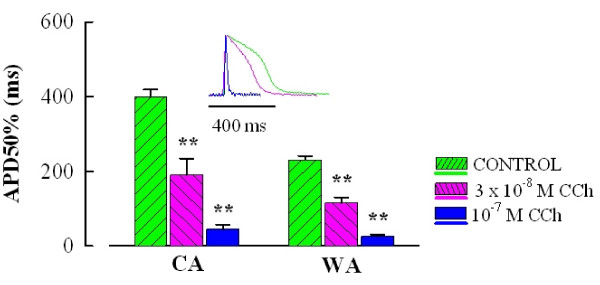
**CCh shortens atrial action potential duration**. The bars show duration of atrial action potential (APD50%) of the crucian carp heart at 50% of repolarization level in the presence of 3·10^-8 ^and 1·10^-7 ^M CCh. The inset shows representative action potential recordings from the warm-acclimated fish heart. The results are means of 10-15 stable impalements from 5 and 3 hearts for CA and WA fish, respectively. Asterisks (**) indicate a statistically significant difference (P < 0.01) from the value in the absence of CCh.

Experiments on sinoatrial preparations suggest that the negative inotropic effect of CCh might be associated with shortening of contraction duration (Figure 1) and therefore caused by a decrease in AP duration. Indeed, intracellular microelectrode recordings in atrial preparations indicate a strong shortening of AP duration by relatively low concentrations of CCh (Figure 3). At the concentration of 3·10^-8 ^M CCh, which inhibits atrial force about 50% but has no effect on sinoatrial pacemaking rate, AP duration was reduced ~50% (48 ± 10 and 50 ± 6% for CA and WA fish, respectively). At 10^-7 ^M CCh, AP duration was curtailed to ~11% of its control value (11 ± 3 and 11 ± 2% for CA and WA fish, respectively). These findings indicate that the decrease in atrial force is closely associated with shortening of AP duration.

**Figure 4:**
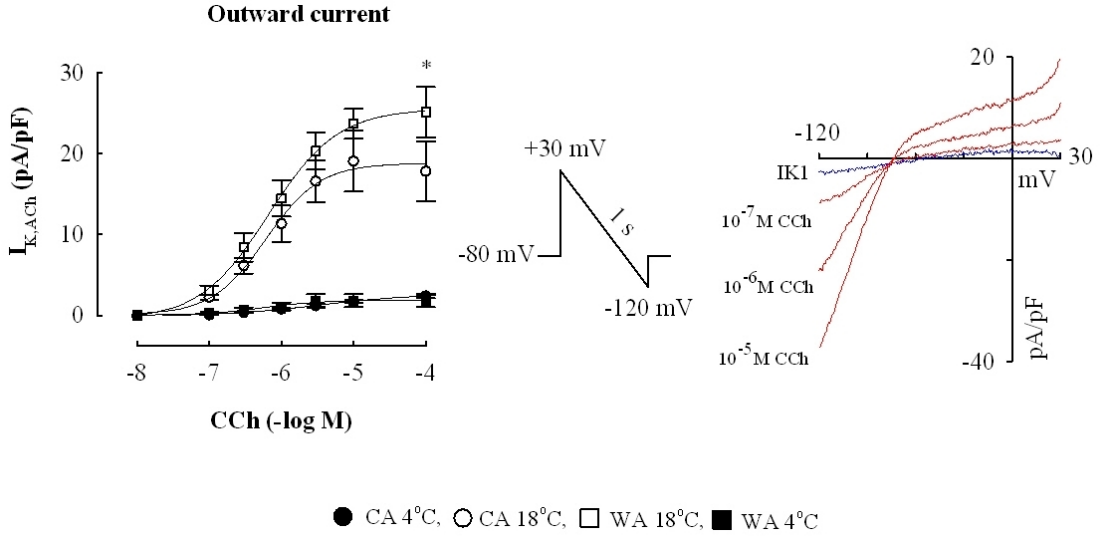
**Activation of the inward rectifier potassium current by CCh**. Induction of the I_K,CCh _by CCh in atrial myocytes from warm-acclimated and cold-acclimated crucian carp heart. Concentration-dependent increases in density of the I_K,CCh _for outward current (left) and representative recordings of the CCh-induced potassium current (red line) from a warm-acclimated atrial myocyte at 18°C, comparison with the background inward rectifier current, I_K1 _(blue line). Note the weak inward rectification of I_K,CCh _in comparison to I_K1. _The experiments were conducted at 4°C and 18°C for both acclimation groups. The results are means ± SEM of 10 myocytes (from 4 fish) for both acclimation groups. Asterisk (*) indicates a statistical difference (P < 0.05) in density of I_K1 _current between cold-acclimated and warm-acclimated fish at 18°C.

CCh induced an inwardly rectifying K^+ ^current, I_K,CCh_, in a concentration-dependent manner (Figure 4). In comparison to the background inward rectifier current (I_K1_), the I_K,CCh _is weakly rectifying and therefore a stronger repolarising current, i.e. it conducts outward current better than the I_K1 _(Figure 4). The threshold concentration for the activation of this current was around 10^-7 ^M for both winter and summer acclimatized fish. The half-maximal activation of I_K,CCh _was attained at the concentration of ~3×10^-7 ^M which is almost an order of magnitude higher than needed to inhibit the force of contraction in sinoatrial preparations (P > 0.05) (Table 2) or to shorten the duration of atrial AP. Thus, opening of the CCh-sensitive inward rectifier K^+ ^channels is a putative mechanism for AP shortening and negative inotropic effect of CCh in atrial muscle, but it is dissociated from atrial force response by a requirement of higher CCh concentrations.

### Effect of CCh on L-type Ca^2+ ^current in atrial myocytes

**Figure 5:**
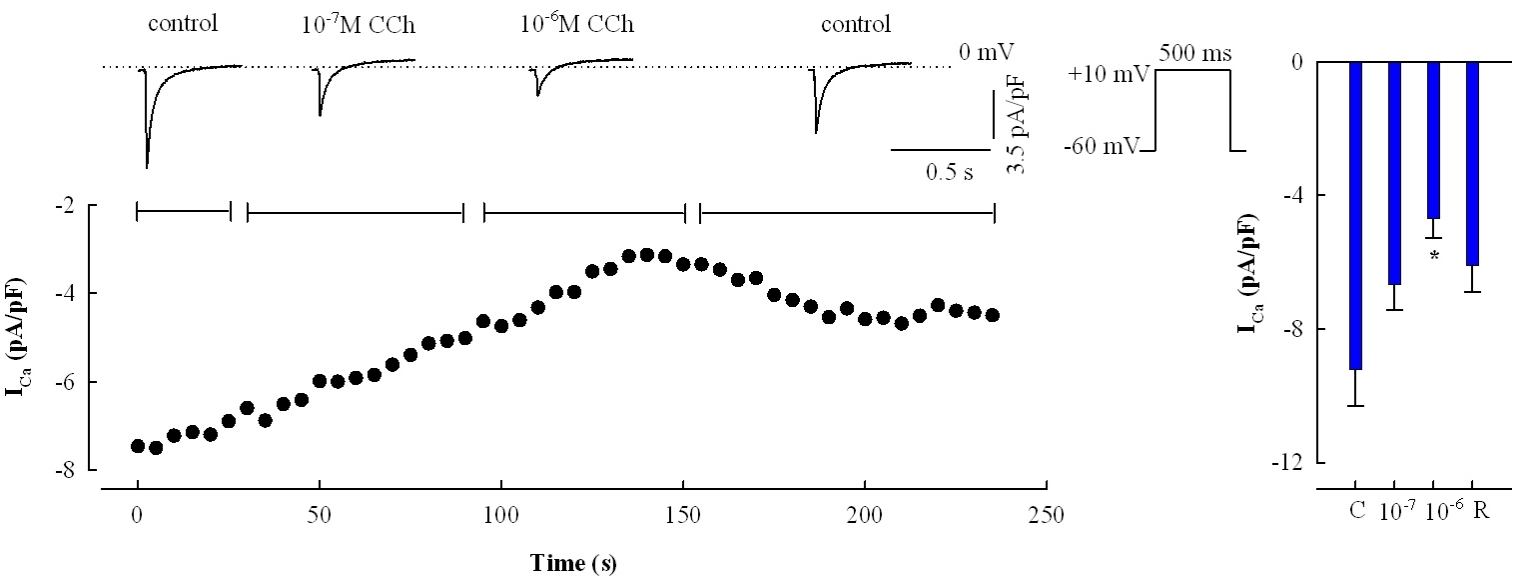
**Inhibition of I_Ca _by CCh**. Effect of 10^-7 ^and 10^-6 ^M CCh on atrial I_Ca _from warm-acclimated and cold-acclimated crucian carp were measured using the whole-cell patch-clamp. (Left) A representative recording showing concentration-dependent inhibition of I_Ca _by CCh and partial recovery (R) of I_Ca _upon washout of CCh. Mean results ± SEM from 12 myocytes (from 3 fish) are shown on the right. CCh induced a small but statistically significant increase of outward current, which disappeared upon washout of CCh. The experiments were conducted on atrial myocytes of warm-acclimated crucian carp at 18°C. Asterisk (*) indicates a statistically significant different value (P < 0.05) from the control (C).

Another possible mechanism of action for CCh is the inhibition of I_Ca _that could also decrease the duration of atrial AP. Effect of CCh on I_Ca _was studied in atrial myocytes of WA fish at 18°C where the current is sufficiently large to discern possible inhibition of I_Ca _by CCh. CCh was applied with a rapid solution changer to minimize the duration of the experiment and thus run-down of I_Ca_. CCh reduced but did not abolish I_Ca _(Figure 5). Inhibition of I_Ca _was 28 ± 8% and 51 ± 6% at the concentration of 10^-7 ^M and 10^-6 ^M CCh, respectively. When CCh was washed out, I_Ca _partly recovered to 66 ± 9% of the initial control value. The inhibitory effect of CCh on I_Ca _suggests that L-type Ca^2+ ^channels of the crucian carp atrial myocytes are phosphorylated under basal conditions and therefore I_Ca _is susceptible to inhibition by muscarinic receptor mediated dephosphorylation. Principally, this effect could contribute to the negative inotropic effect of CCh, but similar to I_K,CCh_, the inhibition of I_Ca _required higher CCh concentrations than were needed to inhibit force production or reduce AP duration in the intact sinoatrial preparations.

**Figure 6:**
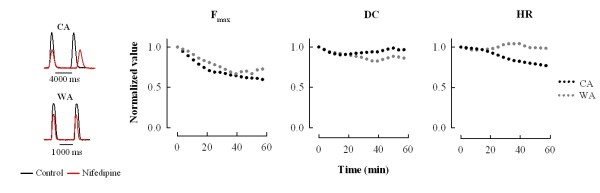
**Depression of atrial contraction force by blockade of I_Ca_**. To estimate the contribution of I_Ca _inhibition to CCh's response in the intact sinoatrial tissue, I_Ca _was specifically blocked with 0.84·10^-6 ^M nifedipine, a concentration that causes about 30% inhibition of I_Ca _i.e. similar I_Ca _inhibition as CCh causes at the concentration of 10^-7 ^M. Representative recordings of contraction from warm-acclimated and cold-acclimated fish hearts (left) and mean responses of force (F_max_), duration of contraction (DC) and heart rate (HR) to nifedipine. Nifedipine causes 34% and 41% inhibition of contractile force for warm-acclimated and cold-acclimated fish, respectively, and slight reduction in duration of contraction. Furthermore, nifedipine slightly depressed heart rate (HR) in cold-acclimated fish but not in warm-acclimated fish. The results are means ± SEM of 6 preparations for both acclimation groups.

Contribution of I_Ca _inhibition to negative inotropic effect of CCh in intact sinoatrial preparations was tested by specifically inhibiting I_Ca _(without activating I_K,CCh_) to the extent it is reduced (~30%) under the maximally effective CCh concentration (10^-7 ^M). To this end, a concentration-dependent inhibition of the atrial I_Ca _by nifedipine (Nif), a specific blocker of L-type Ca^2+ ^channels, was determined (Additional file 1) to find a concentration that produces about 30% inhibition of the current. Blocking about one third of the atrial I_Ca _with 0.84 μM Nif caused 34 ± 8 and 41 ± 9% inhibition of atrial contraction in WA and CA fish, respectively (Figure 6). This suggests that at maximally effective concentration of CCh inhibition of the I_Ca _alone is able to cut about one third of the atrial force.

**Figure 7:**
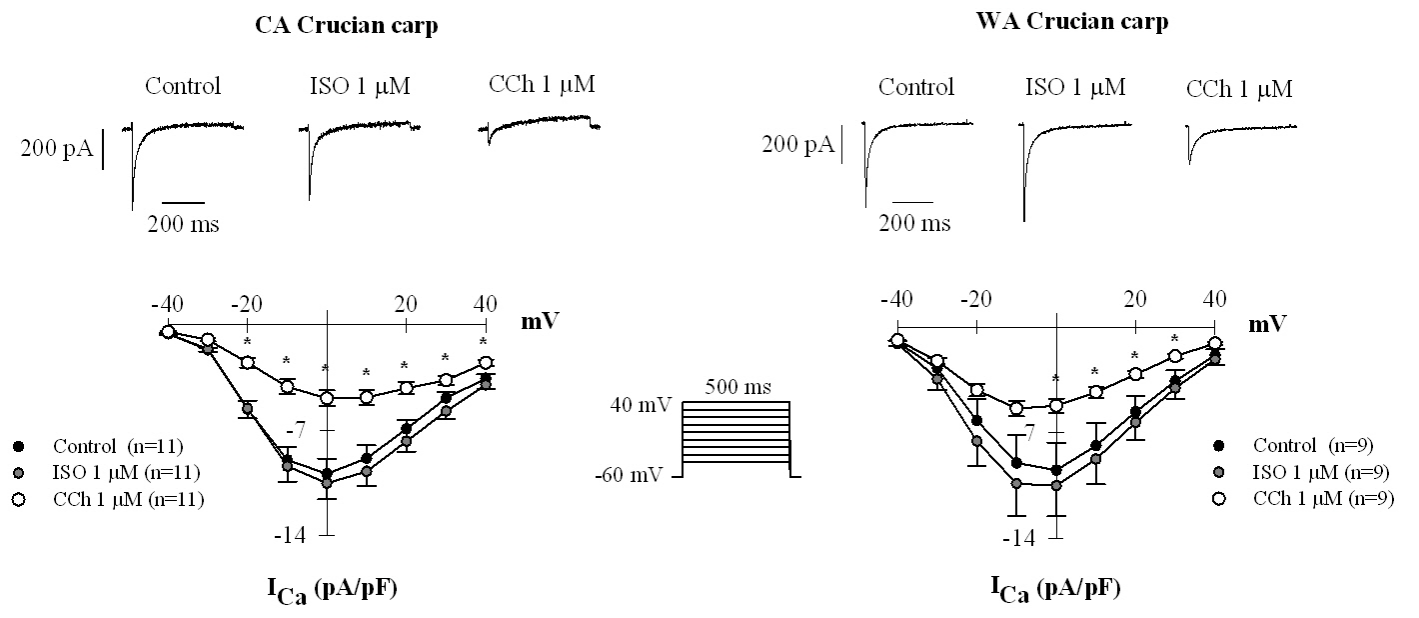
**I_Ca _is not stimulated by Iso**. 10^-6 ^M Iso was rapidly applied to atrial myocytes of warm-acclimated and cold-acclimated crucian carp to see if beta-adrenergic activation could increase I_Ca_. Iso did not enhance I_Ca_. Subsequent exposure of the cells to 10^-6 ^M CCh reduced I_Ca _by about 40%. Experiments were made at 18°C. The results are means ± SEM of 10 myocytes (from 4 fish) for both acclimation groups. Asterisk (*) indicates a statistically significant difference (P < 0.05) between control value and the value in the presence of CCh.

The inhibitory effect of CCh on atrial I_Ca _and the absence of effect by Iso on atrial contraction suggest that L-type Ca^2+ ^channels might be optimally activated under basal conditions. This was tested by measuring the effect of 10^-6 ^M Iso on atrial I_Ca _(Figure 7). The density of I_Ca _in the presence of Iso was 109.7% and 106.3% of the control value (P > 0.05) in WA and CA fish, respectively. Subsequent application of 10^-6 ^M CCh depressed I_Ca _to 50 ± 17 and 57 ± 16% of the control in WA and CA fish, respectively (P < 0.05). These findings suggest that I_Ca _is indeed maximally activated under basal conditions and cannot be further increased by beta-adrenergic activation but can be inhibited by CCh.

## Discussion

The autonomic nervous system represents a major regulatory mechanism for short-term and long-term adjustments of cardiac function via muscarinic cholinergic and beta-adrenergic receptors [16,17]. Seasonal changes in autonomic nervous regulation and neurotransmitter content of the heart has been demonstrated in ectothermic vertebrates [22,23]. Therefore, we hypothesized that autonomic regulation is involved in seasonal acclimatization of the crucian carp heart so that negative cholinergic responses would prevail in winter to down-regulate cardiac function in the cold and anoxic winter waters, while stimulatory adrenergic responses could be stronger in summer to allow enhancement of cardiac activity in warm and oxygen rich waters. The main result in this regard is that little differences in autonomic responses exist between summer and winter fish hearts and therefore the above hypothesis must be abandoned. Obviously, seasonal acclimatization of the crucian carp heart is mainly dependent on intrinsic molecular adjustments within individual cardiac myocytes [3,9,24,25], while sensitivity to extrinsic modulation of cardiac function by the autonomic nervous system remain unaltered throughout the year. Nevertheless, the sinoatrial tissue of crucian carp heart is under a strong inhibitory cholinergic control in summer and in winter.

The present experiments indicate two major findings on autonomic nervous regulation of the crucian carp heart. First, the crucian carp sinoatrial tissue lacks the ability for up-regulation of cardiac activity above the basal level, i.e. there are no stimulatory responses to the beta-adrenergic agonist Iso. Second, atrial force generation is much more sensitive to muscarinic cholinergic inhibition than is firing rate of the sinoatrial pacemaker. CCh concentrations (3·10^-8 ^M - 7·10^-8 ^M) that significantly inhibit atrial force have practically no effect on HR. These characteristics raise questions about the physiological significance of autonomic regulation in the function of crucian carp heart and their molecular mechanisms.

### Cholinergic regulation of the crucian carp heart

**Figure 8:**
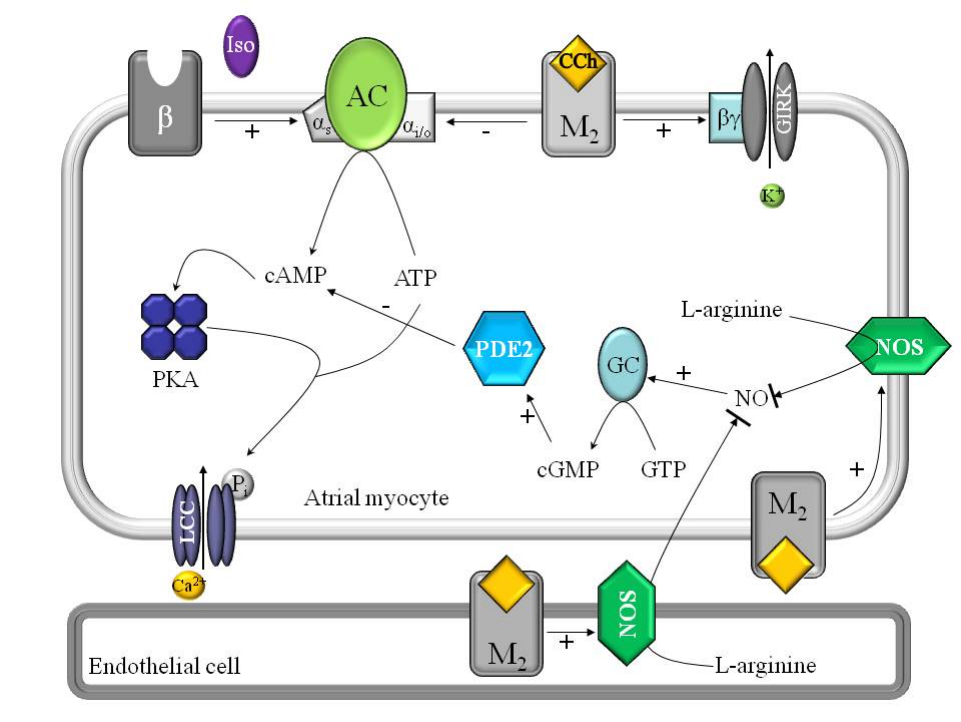
**Putative signaling pathways of autonomic nervous responses**. Muscarinic cholinergic and beta-adrenergic pathways involved in autonomic nervous system responses of crucian carp atrial myocytes. Activation of muscarinic cholinergic receptors induces I_K,CCh _and inhibits I_Ca _via βγ and α_i/o_-subunits of the trimeric G protein, respectively. Inhibition of I_Ca _is dependent on the tonic activation of adenylate cyclase (AC) under basal conditions, i.e. in the absence of agonist binding to beta-adrenergic receptors (β). Nitric oxide synthase (NOS) is not involved in modulating the responses. Cholinergic activation of I_K,CCh _and inhibition of I_Ca _results in shortening of action potential and depression of atrial contraction force. GIRK, G protein activated inward rectifier potassium channel; LCC, L-type calcium channel; M2, muscarinic cholinergic receptor; PDE2, phosphodiesterase; PKA, protein kinase A.

Several teleost fish species, including the goldfish (*Carassius auratus*) a sibling species to crucian carp, have both stimulatory adrenergic and inhibitory cholinergic innervation of the heart which interact via cardiac regulation at the level of adenylyl cyclase [16,20,26,27]. Activation of the sympathetic limb of the autonomic nervous system stimulates myocardial beta-adrenergic receptors and increases adenylyl cyclase activity through the α_s _subunit of the trimeric G protein, whereas increased parasympathetic nerve activity stimulates the muscarinic receptor (M2) that is coupled to inhibition of adenylyl cyclase activity via the α_i/o _subunit of the inhibitory G protein (see Figure 8) [20,21]. These interactions regulate the availability of cAMP to different effectors of the cardiac excitation-coupling, in particular the L-type Ca^2+ ^channels. An additional modulation to cAMP-mediated phosphorylation of L-type Ca^2+ ^channels is produced via nitric oxide synthase (NOS), which is activated via muscarinic receptors and results in cGMP-dependent activation of phosphodiesterase (PDE2) and degradation of cAMP [28-30]. Independent from the cAMP pathway, binding of CCh to the cardiac M2 receptors directly activates an inwardly rectifying K^+ ^current, I_K,CCh_, via the βγ-subunit of the trimeric G protein and shortens AP duration [31](see Figure 8).

Major mechanisms for down-regulation of atrial contractility by the cholinergic system are induction of the I_K,CCh _and inhibition of the I_Ca _[32]. Both mechanisms curtail the duration of AP which therefore indirectly reduce SL Ca^2+ ^influx via Ca^2+ ^channels and the reverse mode of Na^+^-Ca^2+ ^exchange (NCX). Furthermore CCh reduces phosphorylation of the L-type Ca^2+ ^channels and thereby the density of the I_Ca _independently from changes in AP duration [20,21] (see Figure 8). The present experiments on isolated atrial cardiomyocyte show that I_K,CCh _is induced and I_Ca _inhibited by CCh. Hence both mechanisms could potentially contribute to CCh-dependent shortening of AP and consequently to the negative inotropic effect of CCh in the crucian carp heart. However, activation of the currents in isolated myocytes required higher concentrations of CCh than were necessary to induce negative inotropic effects in intact sinoatrial preparations. Therefore changes in I_K,CCh _and I_Ca _do not entirely explain the observed contractile response. This discrepancy might be due to different preparations used for force (intact tissue) and current (isolated myocytes) measurements, in particular due to the presence of endocardial endothelium in the intact tissue which is known to modify cholinergic responses via activation of the endothelial NOS (see Figure 8). However, L-NMMA, an inhibitor of NOS, did not affect CCh responses of the intact sinoatrial tissue, strongly suggesting that NOS activity is not required for the full sensitivity of the atrial tissue to cholinergic agonists in the crucian carp heart. Nitric oxide generation seems to be an obligatory process in cholinergic inhibition of I_Ca _in mammalian pacemaker tissue [32] and endocardial endothelium is found to be necessary for the negative inotropic effect of ACh in the frog (*R. pipiens*) heart [33]. On the other hand, ACh inhibits I_Ca _independent from protein phosphatases or NOS in human atrial tissue [34]. Collectively these findings indicate that contribution of the endocardial NOS to cholinergic responses of the vertebrate heart is species and tissue dependent, and suggest that crucian carp belong to the group which is independent of NOS signaling. However, the presence and activity of NOS and neuropeptides which may modulate autonomic nervous transmission in fish [35] need to be examined with more direct methods in the heart of crucian carp.

The plateau of the cardiac AP is maintained by a balance between small outward K^+ ^currents and a small inward Ca^2+ ^current, and due to the high membrane resistance, fairly small changes in ion conductance can alter AP duration. Hence, only small variations in I_K,CCh _and I_Ca_, which synergistically contribute to the shortening of AP duration during cholinergic activation, are needed to produce a strong shortening of AP duration. A maximally effective concentration of CCh (10^-7 ^M) produces ~30% inhibition of atrial I_Ca _which depresses atrial force with 34-41%. This indirect experimental analysis indicates that CCh-dependent inhibition of I_Ca _alone is capable for almost one third of the CCh's negative inotropic action. Although the same concentration of CCh (10^-7 ^M) activates only a small portion (about 2% of the maximum) of the I_K,CCh,_, the outward I_K,CCh _at 0 mV is 2-3 times larger than the background inward rectifier (I_K1_) and therefore strongly repolarizing. This is because of the weak inward rectification of the I_K,CCh _which makes this current a very effective regulator of AP duration. Thus, full activation of the I_K,CCh _and complete inhibition of the I_Ca _are not needed to produce strong AP shortening and a negative inotropic response. Due to the synergistic action of I_K,CCh _and I_Ca_, it is impossible to provide exact quantitative estimates on the relative importance of I_K,CCh _and I_Ca _in the negative inotropic effect of CCh, even though I_K,CCh _seems to be more important.

An interesting finding is that depression of atrial contraction force occurs prior to the bradycardic response. Almost 80% of the atrial force can be inhibited before CCh's effect on HR starts to appear, i.e. under weak and moderate cholinergic tone atrial contraction is strongly depressed without changes in HR. Similar dichotomy between inotropic and chronotropic effects of cholinergic activation has been noted in the tilapia (*Orechormis nilotica/aureus*) heart [36]. The dissociation of inotropic and chronotropic responses to muscarinic activation probably provides a finely tuned mechanism to regulate cardiac output first by reducing end-diastolic filling of the ventricle via weakened atrial contraction and then via depression of contraction frequency.

### The absence of beta-adrenergic stimulation

The sinoatrial tissue of the crucian carp heart is totally insensitive to beta-adrenergic stimulation lacking both inotropic and chronotropic effects. The absence of these effects is unlikely to result from the research methods or experimental conditions as far as we can routinely measure chronotropic and/or inotropic responses in rainbow trout heart and ventricular muscle of the crucian carp under the same conditions [37,38]. Although cholinergic effects are commonly much stronger than adrenergic effects in teleost hearts, total insensitivity of the fish heart to beta-agonists is, however, rare [15,26]. Ventricular muscle of the crucian carp heart is also fairly insensitive to beta-adrenergic activation, as adrenaline and Iso cause about a modest 10% and 40% increase in the force of contraction, respectively [37]. The absence of beta-adrenergic response in sinoatrial tissue of the crucian carp heart is probably due to maximal or near maximal activation of the molecular machinery involved in beta-adrenergic signaling pathway, in particular the L-type Ca^2+ ^channels, under basal conditions. Phosphorylation of L-type Ca^2+ ^channels due to tonic activation of adenylate cyclase is not uncommon for the vertebrate atrial tissue and may hold true also for the crucian carp atrium. Thus, the present findings suggest that CCh-mediated antagonism of the cAMP-dependent signaling pathway results in dephosphorylation of L-type Ca^2+ ^channels and thereby contribute to the decreased force production (see Figure 8).

Contractility of the crucian carp atrium seems to be tuned high by tonic activation of the adenylate cyclase-cAMP pathway, arguably to increase the scope of cholinergic regulation through the accentuated antagonism of adrenergic and cholinergic mechanisms [17]. In their natural habitat crucian carp are exposed to prolonged winter anoxia. Anoxia has been shown to cause depletion of catecholamine stores of the head kidney in crucian carp [39], possibly due to oxygen requirement of catecholamine synthesis and short half-life of the catecholamine stores [40]. In fish, oxygen deficiency is usually associated with a surge of catecholamines from the chromaffin tissue into the blood for modulation of cardiorespiratory functions and mobilization of the hepatic glycogen reserves [41]. Cardiac stimulation by adrenergic cascade may be useful in hypoxia to optimize oxygen extraction from water, but probably inappropriate in the complete absence of oxygen. In anoxia it would increase cardiac energy consumption in situation where oxygen is not available and when the needs for oxygen convection in the body are abolished. Hence, the unresponsiveness of the crucian carp sinoatrial tissue to adrenergic agonists might serve to prevent unnecessary cardiostimulation upon exposure to complete and prolonged oxygen deficiency.

### Autonomic regulation of HR

Depression of HR required higher CCh concentrations than inhibition of atrial force, suggesting a different mechanism of action. Pacemaker mechanism of the vertebrate heart is an outcome of intricate interactions between several sarcolemmal ion channels, Na^+^-Ca^2+ ^exchange and sarcoplasmic reticulum Ca^2+ ^release [42,43]. Autonomic regulation of the pacemaker rate partly involves the same ionic mechanisms that are needed for regulation of atrial AP, but also additional ion channels like the pacemaker current, I_h _[44]. Muscarinic agonists can cause bradycardia by multiple mechanisms among others through the inhibition of I_h _and I_Ca _or activation of the I_K,CCh _[42,43,45]. The lower sensitivity of the crucian carp pacemaker to CCh in comparison to atrial myocytes may be related to the presence of additional ionic mechanisms of HR regulation, which are absent in atrial myocytes. Alternatively, there may be a lower number of cholinergic receptors in the atrial tissue. These issues cannot be resolved by the current results. Absence of the accelerating effect of Iso on HR suggests that a high constitutive activity of the adenylate-cyclase-cAMP signaling is also present in the pacemaker tissue. However, Stecyk et al. [46] noticed a moderate inhibition of heart rate by a beta-blocker propranolol when applied *in vivo *crucian carp. Whether this discrepancy between the two studies is due to different research methods (tissues and cells versus intact organism) or a genuine difference between larger lake form and smaller-sized pond form of the crucian carp is not known [1].

## Conclusions

Sinoatrial tissue of the crucian carp heart lacks positive chronotropic and inotropic responses to autonomic nervous agonists and contractility can be only down-regulated through activation of the muscarinic cholinergic receptors. In resting conditions, the heart of crucian carp is under vagal tone [46,47], and hence contractility of the sinoatrial tissue can be improved by relief of cholinergic control, even though the stimulatory adrenergic responses are completely absent. The stimulatory signaling pathway of the beta-adrenergic system may exist, but it does not elicit any significant responses in sinoatrial preparations due to the high basal activity of the adenylate cyclase-cAMP signaling. Basal activation of the cAMP pathway provides large scope for up- and down-regulation of contractility by cholinergic mechanisms even in the absence of beta-adrenergic stimulation. The insensitivity of the crucian carp heart to catecholamines prevents unnecessary activation of the heart under oxygen shortage, if hypoxia/anoxia causes a surge of catecholamines into the blood for activation of stress responses, e.g. glycogenolysis in the liver [41]. The high sensitivity of atrial force to CCh suggests that cholinergic activation can cause reduced cardiac output (via reduced ventricular filling) without invoking bradycardia.

Collectively the present findings indicate that responsiveness of the crucian carp heart to extrinsic autonomic modulation is similar in summer and winter acclimatized fish. This is in sharp contrast to the many intrinsic properties of the crucian carp cardiac myocyte which are seasonally primed by environmental cues, in particular by temperature [48], to adjust cardiac function to seasonal changes in oxygen availability and temperature. It remains to be shown, however, whether the cholinergic tone itself is altered by seasons.

## Methods

### Fish

Crucian carp (49.2 ± 12.6 g, n = 83) were caught in June and November from a small local lake. In the laboratory, the fish caught in June and November were maintained at 18°C (warm-acclimated, WA) and 4°C (cold-acclimated, CA), respectively, which are close to the habitat temperatures of those seasons. Therefore throughout the text the terms winter-acclimatized and summer-acclimatized are used synonymous to CA and WA, respectively. Prior to the experiments, the fish were maintained at least four weeks at constant temperature and under a 15 h: 9 h light:dark photoperiod. During that time WA crucian carp were fed three times a week aquarium fish food (Tetra), while no food was provided to CA crucian carp as the winter acclimatized fish fast and deny from food. All experiments were conducted with the consent of the national committee for animal experimentation (permission STH252A).

### Experiments on sinoatrial preparations

For measuring HR and force of atrial contraction, sinoatrial preparations consisting of the sinus venosus and the whole atrium was dissected free and gently fixed with insect pins on a Sylgard-coated 10-ml recording chamber filled with continuously oxygenated (100% O_2_) physiological saline (in mM): 150 NaCl, 3 KCl, 1.2 MgSO_4_, 1.2 NaH_2_PO_4_, 1.8 CaCl_2_, 10 HEPES and 10 glucose adjusted to pH 7.6 with NaOH at 20°C [25,49]. At 4°C pH of the saline was 7.82. The preparation was allowed to equilibrate for about 1 h to reach a stable beating rate before responses to CCh or Iso were determined. Agonist solutions were prepared daily from frozen stocks. Iso solutions included 1 mg/ml ascorbic acid to prevent oxidation of the catecholamine. CCh (10^-10^-3·10^-6 ^M) and Iso (10^-10^-3·10^-5 ^M) were cumulatively added into the bath to construct concentration-response curves. Force signals were digitized (Digidata-1200 AD/DA board Axon Instruments, Saratoga, CA, USA) with a sampling rate of 2 kHz before storing on the computer with the aid of Axotape (Axon Instruments) acquisition software. HR and contractile variables (Figure 1, Table 1) were analyzed with Clampfit software (Axon Instruments) and graphs were constructed in SigmaPlot. The curves were fitted with a Hill equation:(1)

where V_min _is the value of the variable at the highest CCh concentration, V_max _the value of the variable before CCh addition, K_d _the CCh concentration which causes half-maximal effect, [CCh] the CCh concentration, and *H *the Hill slope of the line. Atropine (3·10^-6 ^M) and timolol (3·10^-6 ^M) were used as the blockers of muscarinic cholinergic and beta-adrenergic receptors, respectively. Contractility of crucian carp sinoatrial preparation is stable for at least 3 hours and no corrections for time-dependent deterioration of force and heart rate were made.

### Recording of atrial action potentials

For AP measurements sinoatrial preparations were gently fixed with insect pins on Sylgard-coated bottom of the 10-ml recording chamber filled with oxygenated (100% O_2_) physiological saline. The preparation was allowed to equilibrate for about 1 h to reach a stable beating rate before APs were recorded with sharp microelectrodes. Pipettes were fabricated from borosilicate glass with an internal filament using a Sutter P-97 puller and had a resistance of about 22 MΩs when filled with 3 M KCl. Analog signals were amplified by a high-impedance amplifier (KS-700, WPI, UK) and digitized (Digidata-1200 AD/DA board, Axon Instruments) with sampling rate of 2 kHz before storing on the computer with the aid of Axotape acquisition software [49]. Because of difficulties in getting stable impalements of the cells with microelectrode on moving preparation, AP durations were measured only at three CCh concentrations. Recordings were taken when resting membrane potential was more negative than -60 mV. AP characteristics were analyzed with Clampfit software.

### Measurement of sarcolemmal ion currents using the whole cell patch-clamp method

Single atrial myocytes were isolated by enzymatic digestion using trypsin and collagenase [49,50] and were used within 8 h from isolation.

The whole-cell voltage clamp recording of Ca^2+ ^and K^+ ^currents was performed using an Axopatch 1-D (Axon) and an EPC-9 (HEKA Instruments, Germany) amplifier. A small aliquot of myocyte suspension was transferred to a recording chamber (RC-26; Warner Instrument Corp, Brunswick, CT, USA; volume 150 μl) and superfused with normal K^+^-based saline (containing in mmol l^-1^): 150 NaCl, 5.4 KCl, 1.8 CaCl_2_, 1.2 MgCl_2_, 10 glucose, 10 HEPES, with pH adjusted to 7.6 with NaOH). Temperature of the saline was adjusted to the desired value (4°C or 18°C) with Peltier devices (TC-10 or TC-100, Dagan Corporation, USA) and was continuously monitored with thermocouples positioned closed to the myocytes. Internal perfusion of the myocytes with K^+^-based electrode solution (containing in mmol l^-1^): 140 KCl, 1 MgCl_2_, 5 EGTA, 4 MgATP, and 10 HEPES with pH adjusted to 7.2 with KOH) giving a mean (± SE) pipette resistance of 3.17 ± 0.12 MΩ (n = 62). After gaining access to the cell, pipette capacitance (8.25 ± 0.22 pF) and series resistance (8.49 ± 0.48 MΩ) were compensated. When recording of CCh activated inward rectifier K^+ ^currents (I_K,CCh_), tetrodotoxin (0.5 μM, Tocris Cookson, UK), nifedipine (10 μM, Sigma), E-4031 (1 μM, Alomone labs, Jerusalem, Israel) and glibenclamide (10 μM, Sigma) were always included in the external saline solution to prevent Na^+^, Ca^2+ ^and ATP-sensitive K^+ ^currents, respectively. When recording Ca^2+ ^currents (I_Ca_), KCl was replaced with equimolar CsCl in the bath and pipette solutions. TTX and E-4031 were added to the external saline to prevent Na^+ ^current and Cs^+ ^flux through the delayed rectifier K^+ ^channels, respectively. When measuring I_Ca_, CCh was applied with a fast solution changer (RSC-200, Biologic, Claix, France). Concentration-response curves were fitted with the Hill equation (see *Experiments on sinoatrial preparations*).

## Authors' contributions

The experiments were planned by MV and JH. Experiments with intact sinoatrial preparations were made by MH, MV and JH. JH and MV conducted the electrophysiological experiments. MV is mainly responsible for the writing of the manuscript. All authors have contributed and accepted the final version of the manuscript.

## Supplementary Material

Additional file 1 **Concentration-dependent inhibition of I_Ca _by nifedipine, a specific blocker of L-type Ca^2+ ^channels, in crucian carp atrial myocytes**. To estimate CCh's negative inotropic effect on crucian carp atrial muscle via inhibition of I_Ca_, we sought a nifedipine concentration that causes about 30% inhibition of I_Ca_, i.e. a dose that inhibits I_Ca _to similar extent as 10^-7 ^M CCh. To this end patch-clamp experiments on atrial myocytes of warm-acclimated crucian carp were made at 18°C. Nifedipine was applied to the cell using a rapid solution changer. Two to three concentrations was applied to each cell. Each symbol represents one finding. The results in the figure are from 5 myocytes. Click here for file
