# Supplementary figures and images for: Sinoatrial tissue of crucian carp heart has only negative contractile responses to autonomic agonists
Source: BMC Physiol. 2010 Jun 11;10:10. doi: 10.1186/1472-6793-10-10 (PMC2894799; doi:10.1186/1472-6793-10-10)

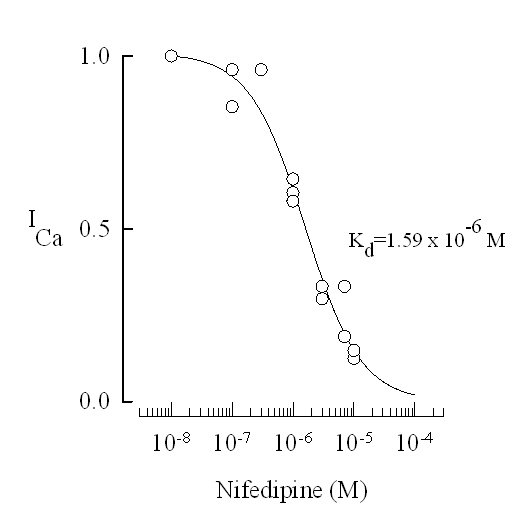

Supplement: Additional file 1 — Concentration-dependent inhibition of ICa by nifedipine, a specific blocker of L-type Ca2+ channels, in crucian carp atrial myocytes. To estimate CCh's negative inotropic effect on crucian carp atrial muscle via inhibition of ICa, we sought a nifedipine concentration that causes about 30% inhibition of ICa, i.e. a dose that inhibits ICa to similar extent as 10-7 M CCh. To this end patch-clamp experiments on atrial myocytes of warm-acclimated crucian carp were made at 18°C. Nifedipine was applied to the cell using a rapid solution changer. Two to three concentrations was applied to each cell. Each symbol represents one finding. The results in the figure are from 5 myocytes. [file 1472-6793-10-10-S1.JPEG]
